# Prognostic Significance of RBM3 Expression in Epithelial Ovarian Cancer: A Tissue Microarray-Based Study

**DOI:** 10.3390/diagnostics15111426

**Published:** 2025-06-03

**Authors:** Hyeong Chan Shin, Hye Won Lee, So-Jin Shin, Sun Young Kwon

**Affiliations:** 1 Department of Pathology, Keimyung University School of Medicine, Daegu 42601, Republic of Korea; chan@dsmc.or.kr (H.C.S.); hwlee@dsmc.or.kr (H.W.L.); 2 Department of Obstetrics and Gynecology, Keimyung University School of Medicine, Daegu 42601, Republic of Korea; sjshinhope2014@gmail.com

**Keywords:** RBM3, ovarian cancer, biomarker, prognosis, immunohistochemistry, survival analysis

## Abstract

**Background/Objectives:** RNA-binding motif protein 3 (RBM3) is a cold-shock protein associated with a favorable prognosis in various malignancies. However, its role in epithelial ovarian cancer (OC) remains unclear. This study aimed to evaluate the prognostic significance of RBM3 expression in OC and its association with clinicopathological features. **Methods:** We retrospectively analyzed 183 cases of OC. Tissue microarrays were constructed using paired 2 mm tumor cores, and RBM3 expression was assessed by immunohistochemistry. Nuclear staining was semi-quantitatively scored based on intensity and proportion, generating a nuclear score (NS). Cases were classified as high (NS > 1) or low (NS ≤ 1) expression. Associations with clinicopathological parameters and survival outcomes were analyzed using chi-square tests, Kaplan–Meier survival curves, and Cox regression models. **Results:** High RBM3 expression was observed in 51.4% of cases and was significantly associated with favorable histologic subtypes (mucinous, endometrioid, clear cell), early International Federation of Gynecology and Obstetrics (FIGO) stage, and the absence of distant metastasis. Subgroup survival analyses stratified by histologic subtype revealed no significant differences in survival outcomes. RBM3 expression was correlated with prolonged disease-free and overall survival, although it did not retain significance in multivariate analysis. **Conclusions:** RBM3 expression is strongly associated with favorable pathological features in epithelial ovarian cancer. Although not an independent prognostic marker, RBM3 may serve as a complementary biomarker for risk stratification and prognosis in clinical practice.

## 1. Introduction

Ovarian cancer (OC) is one of the most common malignancies and ranks as the sixth leading cause of cancer-related death among women in the United States [1]. In Korea, OC is the tenth most common malignancy and the eighth leading cause of cancer-related death among women [2]. The major histological subtypes of OC include serous carcinoma, mucinous carcinoma, endometrioid carcinoma, and clear cell carcinoma. Among various clinical factors, the International Federation of Gynecology and Obstetrics (FIGO) stage has been identified as a key prognostic indicator for OC [3].

RNA-binding motif protein 3 (RBM3) is a cold-shock protein that functions as an RNA-binding molecule involved in post-transcriptional regulation [4]. RBM3 expression is induced under a variety of cellular stress conditions, including mild hypothermia [5], hypoxia [6], and nutrient deprivation [7]. By promoting mRNA stability and enhancing the translation of specific target transcripts, RBM3 contributes to cellular adaptation and survival under stress. Predominantly localized in the nucleus, RBM3 plays a critical role in regulating protein synthesis and maintaining cellular homeostasis [4].

RBM3 has also been explored as a prognostic biomarker in various malignancies. Its expression levels have been associated with tumor differentiation, proliferation, and clinical outcomes [8]. Paradoxically, although RBM3 supports cellular stress resistance and survival, elevated expression has been linked to favorable prognoses in certain cancer types, including breast cancer [9], bladder cancer [10], gastric and esophageal cancers [11], ovarian cancer [12], prostate cancer [13], and malignant melanoma [14].

Although previous studies have reported a correlation between RBM3 expression and clinical outcomes in ovarian cancer, these investigations have been limited by small sample sizes or heterogeneous scoring systems. To date, there remains a lack of large-scale, systematically scored, tissue-based analyses evaluating the prognostic impact of RBM3 in epithelial ovarian cancer. In this study, we sought to address this gap by analyzing RBM3 expression using immunohistochemistry on a well-characterized, single-institution cohort of 183 epithelial ovarian cancer cases, applying a statistically validated nuclear scoring algorithm to stratify RBM3 expression levels. By integrating clinicopathological parameters and survival outcomes, our study aims to provide a more reproducible and clinically applicable assessment of RBM3 as a prognostic marker.

## 2. Materials and Methods

### 2.1. Patients

This study involved 183 patients with OC who underwent surgical resection at Keimyung University Dongsan Hospital between 1998 and 2015. Patients were excluded if they had non-epithelial ovarian tumors, insufficient tumor material, or incomplete clinicopathological or survival data. All tissue samples were fixed in 10% buffered formalin and embedded in paraffin. Patient and tumor characteristics, including age at initial diagnosis, tumor stage, metastasis status, and follow-up data, were obtained from pathology reports and medical records. Tumor staging was classified according to the FIGO system. Overall survival (OS) was defined as the time from diagnosis to death from any cause. Disease-free survival (DFS) was defined as the time from surgical resection to documented disease recurrence, including locoregional or distant metastasis. The requirement for informed consent was waived by the ethics committee. This study was approved by the Institutional Review Board of Dongsan Medical Center (DSMC 2025-04-016).

### 2.2. Construction of Tissue Microarrays

Before TMA construction, all cases were histopathologically reviewed by two board-certified pathologists, Dr. Hyeong Chan Shin and Dr. Sun Young Kwon, using hematoxylin and eosin-stained slides. H&E staining was performed on 4 μm thick sections using the Leica Autostainer XL system with Mayer’s hematoxylin and eosin Y, followed by dehydration, clearing, and coverslipping. Any discrepancies were resolved by consensus. For each case, a representative tumor block was selected based on a high tumor cell density, minimal necrosis, and well-preserved morphology. Regions containing characteristic histologic features of the tumor subtype were prioritized to ensure consistent sampling. A pair of 2 mm tissue cores was extracted from each donor block and transferred to a recipient block using the Quick-Ray Manual Tissue Microarrayer (Unitma, Seoul, Republic of Korea). Normal ovarian tissue was not included in the TMA. All TMAs were prepared by the Department of Pathology, Keimyung University Dongsan Hospital. All 183 cases were successfully sampled using paired 2 mm tumor cores, and no cores were lost or excluded during TMA construction or staining.

### 2.3. Immunohistochemical Assessment and Scoring

IHC was performed using the automated Benchmark platform (Ventana Medical Systems, Tucson, AZ, USA) following the manufacturer’s protocol. Four-micrometer-thick tissue sections were stained for RBM3 using the UltraView™ Universal DAB detection kit (Ventana). Antigen retrieval was conducted with cell conditioning solution (CC1, Ventana), and endogenous peroxidase activity was blocked. Immunohistochemical staining for RBM3 was performed using a rabbit polyclonal anti-RBM3 antibody (Sigma-Aldrich, catalog no. HPA003624, St. Louis, MO, USA) at a dilution of 1:100. Hematoxylin counterstaining was performed using Mayer’s Hematoxylin (Dako, catalog no. S3309, Carpinteria, CA, USA). Slides were subsequently dehydrated, cleared, and coverslipped.

RBM3 protein expression was predominantly nuclear in tumor cells. The nuclear fraction (NF) was categorized as follows: 0 (0–1%), 1 (2–25%), 2 (26–50%), 3 (51–75%), and 4 (>75%). Nuclear intensity (NI) was scored as 0 (negative), 1 (weak), 2 (moderate), or 3 (strong). The nuclear score (NS) was calculated by multiplying NF and NI, yielding a range of 0 to 12. For statistical analysis, RBM3 expression was classified as low (NS ≤ 1) or high (NS > 1), based on the heterogeneity in OS assessed by the log-rank test.

To minimize the variability related to long-term tissue storage and IHC interpretation, all samples were fixed in 10% buffered formalin shortly after surgical resection and embedded using standardized protocols. RBM3 immunostaining was performed in a single laboratory using the same antibody lot and detection system, with standardized antigen retrieval conditions. External positive control tissues were included in each staining batch. All immunostained slides were independently interpreted by two board-certified pathologists, Dr. Hyeong Chan Shin and Dr. Sun Young Kwon. Any discrepancies in RBM3 scoring were resolved by consensus to ensure consistency and minimize interobserver variability. Staining quality and tissue preservation were verified through a histological review of H&E slides, and RBM3’s nuclear localization pattern was consistent with known expression profiles. No additional internal nuclear or membrane reference markers were used.

### 2.4. Statistical Analyses

Statistical analyses were conducted using SPSS version 20.0 for Windows (IBM Corp., Armonk, NY, USA). Associations between RBM3 expression and clinicopathological variables were assessed using the chi-square test. Kaplan–Meier survival curves were generated for OS and DFS, and differences were assessed using the log-rank test. Cox proportional hazards regression models were used to identify independent prognostic factors. Hazard ratios (HRs) and 95% confidence intervals (CIs) were reported. Differences in the age distribution across histologic subtypes and FIGO stages were evaluated using the Kruskal–Wallis test. A *p*-value of <0.05 was considered statistically significant.

## 3. Results

### 3.1. Patient Characteristics and Histological Features

The study cohort consisted of 183 patients with OC. The median age was 53 years (range: 11–79). The most common histologic subtype was serous carcinoma (57.4%), followed by clear cell (17.5%), endometrioid (15.8%), and mucinous carcinoma (9.3%). FIGO stage 3 tumors comprised the largest group (55.7%), while grades 1, 2, and 4 accounted for 33.3%, 9.3%, and 1.6%, respectively. Distant metastasis was present in 57.4% of patients, and 32.8% experienced recurrence. At the time of last follow-up, 33.3% of patients had died. These baseline characteristics are summarized in Table 1.

### 3.2. RBM3 Expression and Clinicopathological Correlations

**Figure 1:**
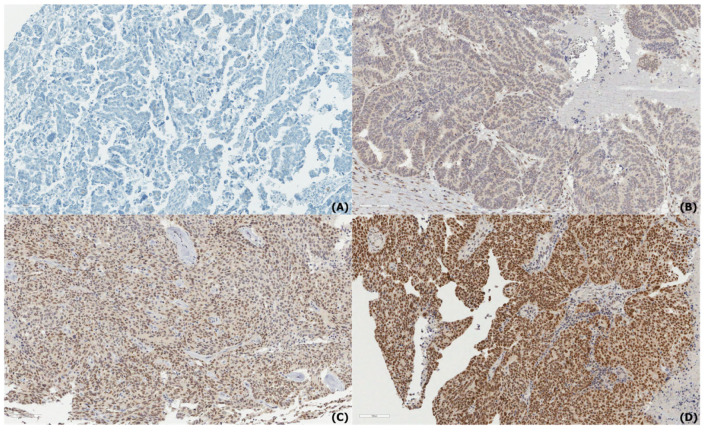
Immunohistochemical staining of RNA-binding motif protein 3 (RBM3) in serous carcinoma. (**A**) Negative expression, (**B**) Weak expression, (**C**) Moderate expression, and (**D**) Strong expression of RBM3. All images were captured at ×200 magnification; scale bar = 100 μm (visible in the lower left corner of each image). Representative images were selected based on pathologist-reviewed H&E slides. Stage- or subtype-specific comparisons are not performed due to a lack of paired image documentation.

**Figure 2:**
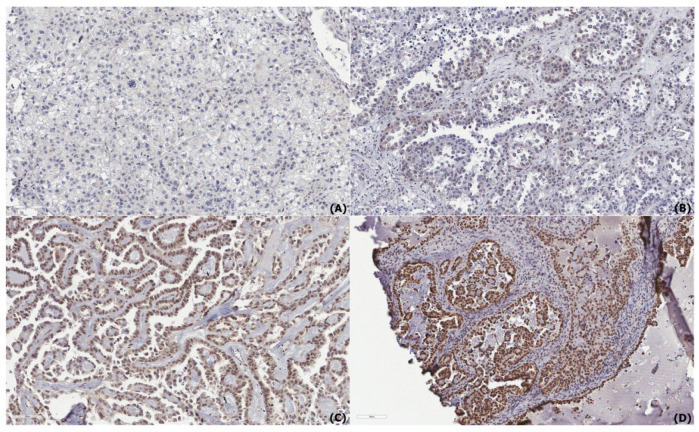
Immunohistochemical staining of RNA-binding motif protein 3 (RBM3) in clear cell carcinoma. (**A**) Negative expression, (**B**) Weak expression, (**C**) Moderate expression, and (**D**) Strong expression of RBM3. All images were captured at ×200 magnification; scale bar = 100 μm (visible in the lower left corner of each image). Representative images were selected based on pathologist-reviewed H&E slides. Stage- or subtype-specific comparisons are not performed due to a lack of paired image documentation.

To evaluate the association between nuclear RBM3 expression and clinicopathological features, the IHC results from the TMAs were analyzed. Representative images of the RBM3 expression intensity (negative to strong) in serous (Figure 1) and clear cell carcinoma (Figure 2) are shown. A summary of the clinicopathological parameters and RBM3 expression levels is provided in Table 2. Among the 183 OC cases, high nuclear RBM3 expression (NS > 1) was observed in 94 cases (51.4%), while 89 cases (48.6%) demonstrated low expression (NS ≤ 1). High RBM3 expression was more commonly observed in younger patients, although the association was not statistically significant (*p* = 0.104).

Based on chi-square analysis, significant associations were identified between RBM3 expression and the histologic subtype (*p* < 0.001), FIGO stage (*p* = 0.003), and the presence of distant metastasis (*p* = 0.002). High RBM3 expression was most frequently observed in mucinous carcinoma (88.2%), followed by clear cell carcinoma (71.9%), endometrioid carcinoma (69.0%), and serous carcinoma (34.3%). Additionally, tumors with high RBM3 expression were more often classified as lower FIGO stages. In contrast, no significant associations were found between RBM3 expression and recurrence (*p* = 0.637) or mortality status at last follow-up (*p* = 0.117) (Table 2).

**Figure 3:**
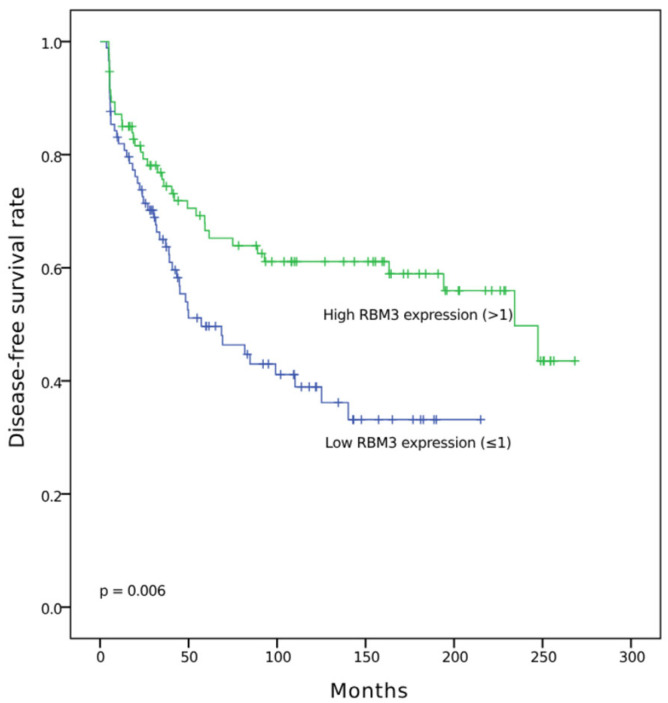
Kaplan–Meier curve for disease-free survival in ovarian cancer according to RBM3 expression (high vs. low).

**Figure 4:**
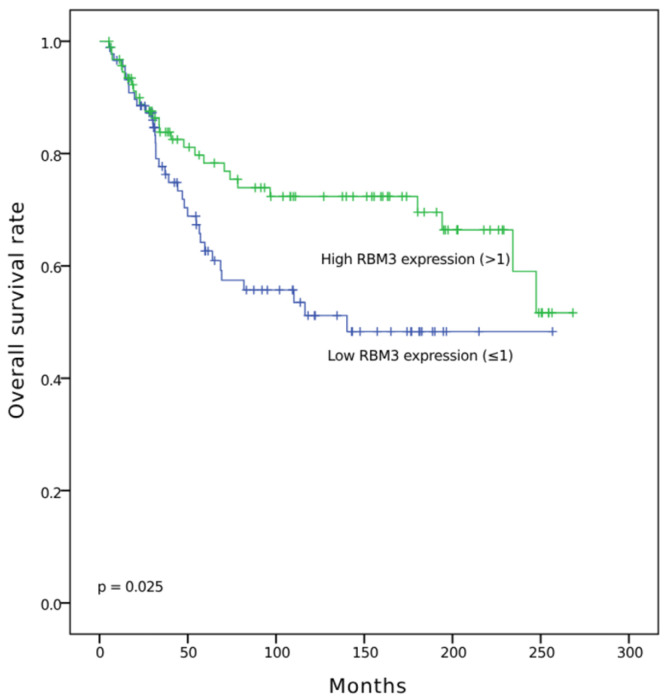
Kaplan–Meier curve for overall survival in ovarian cancer according to RBM3 expression (high vs. low).

To evaluate the prognostic impact of RBM3 expression levels in OC, OS, and DFS, Kaplan–Meier analysis was performed. Subgroup analyses stratified by histologic subtype (serous, endometrioid, clear cell, and mucinous) were also conducted. Kaplan–Meier survival curves for DFS and OS according to RBM3 expression were generated for each subtype, but no statistically significant differences were observed. These results are provided in Supplementary Figure S1. FIGO stage-specific survival curves were not analyzed in this study. High nuclear RBM3 expression was significantly associated with longer DFS (*p* = 0.006) (Figure 3) and OS (*p* = 0.025) (Figure 4) compared to low expression.

Univariate analysis revealed that high RBM3 expression, age, histologic subtype, FIGO stage, and metastasis status were significantly associated with both DFS and OS. However, in multivariate analysis, the FIGO stage remained an independent prognostic factor for both DFS and OS. Additionally, age was identified as an independent prognostic factor for OS (Table 3 and Table 4).

We further evaluated the relationship between patient age and tumor subtype or FIGO stage. Kruskal–Wallis tests demonstrated statistically significant differences in the age distribution across histologic subtypes (*p* = 0.021) and FIGO stages (*p* = 0.010). These findings suggest that older age may be associated with specific cancer types and more advanced disease stages in this cohort.

## 4. Discussion

In this study, we evaluated the prognostic significance of RBM3 expression in OC using IHC staining on a TMA cohort. Our findings demonstrated that high nuclear RBM3 expression was significantly associated with prolonged DFS and OS. However, the multivari-ate analysis identified FIGO stage as the only independent prognostic factor for both DFS and OS, with age also emerging as an independent prognostic factor for OS. Although RBM3 did not emerge as an independent prognostic factor in multivariate analysis, its strong association with early-stage disease, the absence of distant metastasis, and favorable histologic subtypes highlights its potential utility as a contextual biomarker in clinical practice. This trend was also reflected morphologically, as tumors with a lower FIGO stage or specific histologic subtype often demonstrated a stronger RBM3 nuclear staining intensity, in line with the statistical correlations observed. While we did not include separate image panels comparing RBM3 expression across FIGO stages or histologic subtypes, all representative images were selected based on the pathologist-reviewed H&E morphology to reflect a range of expression intensities within major histologic subtypes. Particularly in settings where comprehensive molecular profiling is unavailable, RBM3 expression—assessed via routine immunohistochemistry—may aid in refining risk stratification, especially among patients with histologically indolent tumors.

RBM3 has previously been explored as a prognostic biomarker in multiple malignancies, including breast cancer [9], bladder cancer [10], gastric and esophageal cancers [11], OC [12], prostate cancer [13], and malignant melanoma [14]. Consistent with earlier studies, our results support the association between high RBM3 expression and favorable clinical outcomes in OC patients. Despite this, large-scale, comprehensive evaluations of RBM3 in OC are limited, underscoring the contribution of our study to the existing body of literature.

RBM3 is a cold-shock protein that facilitates cellular adaptation and survival under stress conditions such as hypoxia [6] and nutrient deprivation [7], primarily by maintaining cellular homeostasis. Although this pro-survival function might appear contradictory in the oncologic context, where enhanced cell survival could promote tumor progression, elevated RBM3 expression has been consistently associated with improved clinical outcomes. A potential explanation is that RBM3 may contribute to maintaining a more differentiated tumor phenotype, which is typically associated with less aggressive behavior and favorable prognosis. RBM3 also modulates several biological processes relevant to cancer, including apoptosis [15], DNA repair [16], and cell proliferation [17], thereby influencing tumor behavior and its response to treatment. Moreover, RBM3 regulates post-transcriptional events such as mRNA stability and protein synthesis, processes that may suppress tumor aggressiveness or promote cellular differentiation [4]. Given the recognized heterogeneity of ovarian cancer, we further conducted subgroup analyses stratified by histologic subtype to evaluate the prognostic value of RBM3 within individual tumor types. Kaplan–Meier survival analyses for serous, clear cell, endometrioid, and mucinous carcinomas revealed no statistically significant differences in DFS or OS according to the RBM3 expression status (Supplementary Figure S1). These findings suggest that, while RBM3 is associated with favorable clinicopathological features in the overall cohort, its prognostic impact may not be uniformly maintained across all histologic subtypes. The limited sample sizes within non-serous groups may also reduce the statistical power required to detect meaningful survival differences. Nonetheless, these results underscore the importance of considering tumor subtype in the interpretation of biomarker data. Furthermore, our study did not incorporate molecular classification schemes such as those defined by The Cancer Genome Atlas (TCGA), which distinguish transcriptomic subtypes with distinct clinical and prognostic features. Future investigations integrating molecular stratification may help elucidate whether RBM3’s expression correlates with specific genomic or transcriptomic subtypes of ovarian cancer and clarify its utility as a subtype-specific biomarker. These findings indicate that RBM3 may exert subtype-specific roles in OC progression and therapeutic responsiveness.

Emerging evidence also points to a potential role for RBM3 in modulating oncogenic signaling pathways, including the PI3K/AKT/mTOR and MAPK pathways [18]. Further studies are warranted to understand its mechanistic involvement in tumor progression and chemoresistance.

RBM3 may also interact with the tumor microenvironment [19], particularly through the modulation of immune cell infiltration and inflammatory responses. Its involvement in immune evasion mechanisms, potentially influencing tumor-associated macrophages and regulatory T cells, suggest that RBM3 may influence immunotherapy outcomes. Future studies should explore the correlation between RBM3 expression and immune checkpoint markers, such as Programmed Death-Ligand 1, to assess its predictive potential for an immunotherapy response in OC.

Beyond its prognostic value, RBM3 may serve as a predictive biomarker for treatment response. Previous studies have associated high RBM3 expression with an increased sensitivity to chemotherapy in other cancer types [12,20]. Further investigation is needed to determine whether RBM3 expression can stratify OC patients according to their responsiveness to standard treatments. Prospective clinical trials incorporating RBM3 expression analysis may yield clinically relevant insights and contribute to more personalized therapeutic strategies.

Despite the strengths of this study, several limitations must be acknowledged. First, this was a retrospective, single-institution study, which may limit the generalizability of the findings. Validation in larger, multi-institutional cohorts is necessary. In addition, the retrospective design and single-institution setting may have introduced selection bias related to patient population or clinical practices. Second, while IHC remains a widely used technique for protein expression analysis, it is inherently semi-quantitative and subject to interobserver variability. The incorporation of digital pathology or alternative quantitative techniques, such as RT-PCR or Western blotting, may offer a more accurate assessment of RBM3 expression. In particular, the integration of machine learning-based image analysis or fully automated scoring systems in digital pathology platforms may help reduce interobserver variability and enhance reproducibility across institutions. Third, we did not include detailed treatment-related variables such as the type of chemotherapy regimen, number of treatment cycles, or therapeutic response. These data were not consistently available for all patients in this retrospective cohort and were therefore excluded from our analysis. Given that such treatment factors may significantly influence survival outcomes, their omission limits our ability to evaluate RBM3’s predictive value in the context of therapy. Future studies with comprehensive treatment data are necessary to clarify the potential role of RBM3 as a predictive biomarker. Fourth, while we conducted subgroup analyses by histologic subtype, RBM3 expression was not significantly associated with DFS or OS in any individual subtype. These negative findings may reflect the insufficient statistical power due to the small sample sizes in non-serous groups. Additionally, our analysis did not incorporate molecular classifications such as TCGA-defined transcriptomic subtypes, which may provide more refined prognostic insights. Future studies integrating molecular stratification and larger subtype-specific cohorts are warranted to clarify RBM3’s prognostic role within specific tumor subtypes.

The cutoff value used to distinguish high and low RBM3 expression was determined statistically, which may introduce variability across different patient populations. This highlights the need for standardization in RBM3 assessment, possibly through machine learning-assisted image analysis or standardized scoring algorithms, which could improve reproducibility and clinical applicability. In previous studies, cutoff values have varied depending on the scoring system and cancer type [9,10,11,12,13,14,20,21]. In contrast, our study adopted a relatively low cutoff (NS > 1), based on the statistical heterogeneity in overall survival, similar to thresholds applied in breast cancer using the Allred scoring system [22]. Both the RBM NS in OC and the Allred score in breast cancer define low expression as weak intensity in less than 1% of tumor cells. The Allred score, which combines the proportion and intensity of nuclear staining, frequently categorizes low expression as scores ≤ 2, aligning with our definition of low RBM3 expression (NS ≤ 1). This approach enabled the sensitive discrimination of survival outcomes but reinforces the importance of developing cancer-specific, standardized scoring algorithms to enhance reproducibility and cross-study comparability. Future studies should aim to validate this cutoff in independent cohorts and leverage machine learning-assisted image analysis to reduce observer-dependent variability.

Our findings suggest that RBM3 may serve as a useful prognostic biomarker in OC, in relation to DFS and OS. However, due to its lack of independent prognostic value in multivariate analysis, RBM3 is unlikely to function effectively as a standalone biomarker. Rather, it may augment existing clinicopathological factors in enhancing prognostic accuracy.

Future studies should focus on elucidating the molecular mechanisms underlying RBM3’s role in OC progression. Functional experiments are needed to clarify RBM3’s role in chemoresistance, metastasis, and tumor microenvironment interactions. Additionally, prospective clinical trials incorporating RBM3 expression analysis may determine its value as a predictive biomarker for treatment response, ultimately guiding personalized therapeutic strategies in OC patients.

Investigating RBM3 as a potential therapeutic target also warrants attention. If RBM3 modulates tumor behavior through specific signaling pathways, the pharmacologic targeting of its activity may represent a novel therapeutic strategy in OC. The interplay between RBM3 and key oncogenic pathways, such as the PI3K/AKT/mTOR or MAPK pathways, should be further explored.

Compared to previous studies that have reported the prognostic relevance of RBM3 in ovarian cancer, our study adds value by utilizing one of the largest TMA-based cohorts to date, applying a statistically validated cutoff scoring system, and focusing on a reproducible nuclear scoring method. Although RBM3 did not emerge as an independent prognostic factor in multivariate analysis, its strong association with favorable histological subtypes and early-stage disease highlights its potential as an adjunctive biomarker. Specifically, RBM3 expression may aid in refining risk stratification models and guide prognostic assessment in patients with histologically favorable or early-stage epithelial ovarian cancer, particularly where advanced molecular testing is not readily available. Although RBM3 can exhibit cytoplasmic localization in certain contexts, immunohistochemical evaluation in this study revealed predominantly nuclear staining in ovarian carcinoma tissues. Cytoplasmic expression was not consistently observed and was therefore not included in the scoring algorithm. This approach aligns with prior studies in other tumor types, such as breast and prostate cancers, which also focused on nuclear RBM3 expression. According to the Human Protein Atlas (www.proteinatlas.org), RBM3 shows primarily nuclear localization in most malignancies, including ovarian cancer [23]. Future investigations using multiplex immunohistochemistry or subcellular fractionation may help clarify the clinical significance of cytoplasmic RBM3 expression.

## 5. Conclusions

High RBM3 expression was significantly associated with favorable clinicopathological characteristics, including a low FIGO stage, absence of distant metastasis, and a favorable histologic subtype in epithelial ovarian cancer. Patients with high RBM3 expression also demonstrated prolonged disease-free and overall survival. While RBM3 expression alone may not function as an independent prognostic marker, our findings support its relevance as a complementary biomarker that reflects favorable biological behavior in epithelial ovarian cancer. Incorporating RBM3 immunoreactivity into diagnostic workflows could assist in identifying patients with less aggressive disease who may benefit from tailored follow-up strategies. Further multicenter validation and mechanistic studies are warranted to clarify RBM3’s functional role and therapeutic potential in ovarian cancer.

## Figures and Tables

**Table 1:** Clinicopathological characteristics of the study cohort (*n* = 183).

Characteristic	Number (%)
Age (year)	53 years (11–79)
Histologic subtype	
Serous carcinoma	105 (57.4)
Mucinous carcinoma	17 (9.3)
Endometrioid carcinoma	29 (15.8)
Clear cell carcinoma	32 (17.5)
FIGO stage	
1	61 (33.3)
2	17 (9.3)
3	102 (55.8)
4	3 (1.6)
Distant metastasis	
Absent	78 (42.6)
Present	105 (57.4)
Recurrence	
Absent	123 (67.2)
Present	60 (32.8)
Expire	
No	122 (66.7)
Yes	61 (33.3)

**Table 2:** Correlation of RBM3 expression with clinicopathologic characteristics of ovarian carcinomas.

Characteristic	RBM3 Expression		*p*-Value
	Low Expression No. (%)	High Expression No. (%)	
Age (year)	54.62 (25–75)	51.56 (20–78)	0.104
Histologic subtype			<0.001
Serous carcinoma	69 (65.7)	36 (34.3)	
Mucinous carcinoma	2 (11.8)	15 (88.2)	
Endometrioid carcinoma	9 (31.0)	20 (69.0)	
Clear cell carcinoma	9 (28.1)	23 (71.9)	
FIGO stage			0.003
1	21 (34.4)	40 (65.6)	
2	7 (41.2)	10 (58.8)	
3	59 (57.8)	43 (42.2)	
4	2 (66.7)	1 (33.3)	
Distant metastasis			0.002
Absent	27 (34.6)	51 (65.4)	
Present	62 (59.0)	43 (41.0)	
Recurrence			0.637
Absent	58 (47.2)	65 (52.8)	
Present	31 (51.7)	29 (48.3)	
Expire			0.117
No	54 (44.3)	68 (55.7)	
Yes	35 (57.4)	26 (42.6)	

**Table 3:** Univariate analysis of DFS and OS in ovarian carcinomas.

Characteristic	DFS		*p*-Value	OS		*p*-Value
	HR	95% CI		HR	95% CI	
Age	1.023	1.005–1.041	0.011	1.041	1.018–1.065	<0.001
Histologic subtype						
(Non-serous vs. serous)	3.648	2.163–6.155	<0.001	2.641	1.478–4.720	0.001
FIGO stage						
(Stage 1,2 vs. Stage 3,4)	10.627	5.580–20.240	<0.001	13.066	5.572–30.636	<0.001
Metastasis						
(Absent vs. present)	7.454	4.093–13.573	<0.001	6.972	3.411–14.254	<0.001
RBM3 expression						
(High vs. Low)	0.547	0.352–0.849	0.007	0.558	0.333–0.937	0.027

**Table 4:** Multivariate analysis of DFS and OS in ovarian carcinomas.

Characteristic	DFS		*p*-Value	OS		*p*-Value
	HR	95% CI		HR	95% CI	
Age	1.002	0.983–1.022	0.053	1.03	1.004–1.056	0.021
Histologic subtype						
(Non-serous vs. serous)	1.201	0.602–2.398	0.604	0.783	0.383–1.598	0.501
FIGO stage						
(Stage 1,2 vs. Stage 3,4)	10.627	5.580–20.240	<0.001	14.346	4.316–47.684	<0.001
Metastasis						
(Absent vs. present)	1.113	0.371–3.335	0.849	0.813	0.276–2.398	0.708
RBM3 expression						
(High vs. Low)	0.894	0.562–1.422	0.635	0.777	0.444–1.360	0.377

## Data Availability

The datasets used and/or analyzed during the current study are available from the corresponding author on reasonable request.

## Supplementary Materials

The following supporting information can be downloaded at: https://www.mdpi.com/article/10.3390/diagnostics15111426/s1, Figure S1: Kaplan–Meier survival curves for disease-free survival (DFS; left) and overall survival (OS; right) stratified by RBM3 expression status in ovarian cancer subtypes. Subtypes include: (A,B) serous carcinoma, (C,D) clear cell carcinoma, (E,F) endometrioid carcinoma, and (G,H) mucinous carcinoma. No statistically significant survival differences were observed in any subtype (all *p* > 0.05).

## Abbreviations

The following abbreviations are used in this manuscript:

RBM3	RNA-binding motif protein 3
OC	ovarian cancer
DFS	disease-free survival
OS	overall survival
TMA	tissue microarray
IHC	immunohistochemistry
FIGO	International Federation of Gynecology and Obstetrics
