# Supplementary material for: Prognostic Significance of RBM3 Expression in Epithelial Ovarian Cancer: A Tissue Microarray-Based Study
Source: Diagnostics (Basel). 2025 Jun 3;15(11):1426. doi: 10.3390/diagnostics15111426 (PMC12155555; doi:10.3390/diagnostics15111426)
Supplement: Supplementary file 1 [file diagnostics-15-01426-s001.zip › diagnostics-3624864-supplementary.pdf]

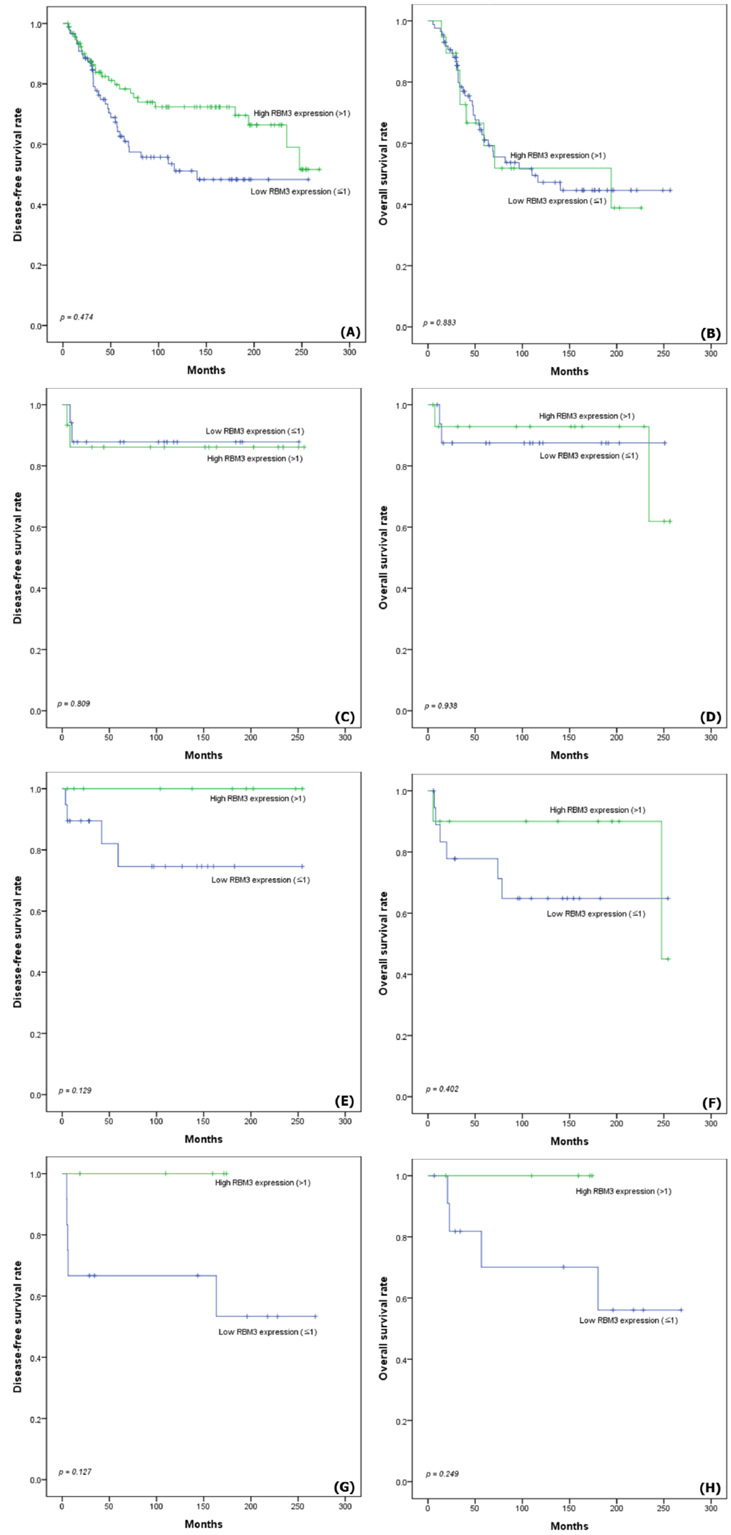

Supplementary Figure S1. Kaplan–Meier survival curves for disease-free survival (DFS; left) and overall survival (OS; right) stratified by RBM3 expression status in ovarian cancer subtypes. Subtypes include: (A,B) serous carcinoma, (C,D) clear cell carcinoma, (E,F) endometrioid carcinoma, and (G,H) mucinous carcinoma. No statistically significant survival differences were observed in any subtype (all $p > 0.05$).
